# Early Gastric Cancer with Purely Enteroblastic Differentiation and No Conventional Adenocarcinoma Component

**DOI:** 10.1155/2018/3620293

**Published:** 2018-08-28

**Authors:** Rin Yamada, Shin-ichiro Horiguchi, Tomoko Onishi, Toru Motoi, Tsunekazu Hishima

**Affiliations:** ^1^Department of Pathology, Tokyo Metropolitan Cancer and Infectious Diseases Center, Komagome Hospital, 3-18-22, Honkomagome, Bunkyo-ku, Tokyo 113-8677, Japan; ^2^Department of Gastroenterology, Tokyo Metropolitan Cancer and Infectious Diseases Center, Komagome Hospital, 3-18-22, Honkomagome, Bunkyo-ku, Tokyo 113-8677, Japan

## Abstract

Gastric carcinoma with enteroblastic differentiation (GCED) is a rare variant of gastric carcinoma, and a part of GCED produces alpha-fetoprotein. GCED is characterized by cells with clear cytoplasm and a tubulopapillary and solid growth pattern resembling those in the primitive gut. GCED is typically overlaid by a conventional adenocarcinoma (CA) component, implying that CA in the mucosa differentiates into GCED during tumor invasion and proliferation. We present the case of a 73-year-old woman with a 10-mm superficial elevated lesion and a slight central depression at the anterior wall of the lower gastric body. Endoscopic submucosal dissection revealed tumor cells having clear cytoplasm and severely atypical nuclei characteristic of GCED. The growth pattern was predominantly solid and trabecular but included submucosal layer invasion and limited tubular growth. Atrophic pyloric mucosa without intestinal metaplasia surrounded the tumor. Immunohistochemically, the tumor cells were positive for AFP, GPC3, and SALL4. The present patient showed a purely enteroblastic differentiation without a CA component despite the presence of early cancer, indicating that few cases of GCED may arise de novo in the gastric mucosa. GCED is more aggressive compared with CA; therefore, pathologists should be aware that GCED without CA can appear in biopsy specimens of early cancer while making an accurate diagnosis.

## 1. Introduction

Gastric carcinoma with enteroblastic differentiation (GCED) is a rare variant of gastric carcinoma, and a part of GCED produces alpha-fetoprotein (AFP) [1–4]. Cytologically, GCED is characterized by clear cytoplasm. Histologically, it is characterized by a tubulopapillary and solid growth pattern resembling the primitive gut; additionally, GCED is usually overlaid by a conventional adenocarcinoma (CA) component, which suggests that CA in the mucosa differentiates into GCED during the process of tumor invasion and proliferation [1–4]. Here we present a very rare case of a patient with GCED, which demonstrated a purely enteroblastic differentiation without a CA component despite the presence of early cancer.

## 2. Case Presentation

**Figure 1:**
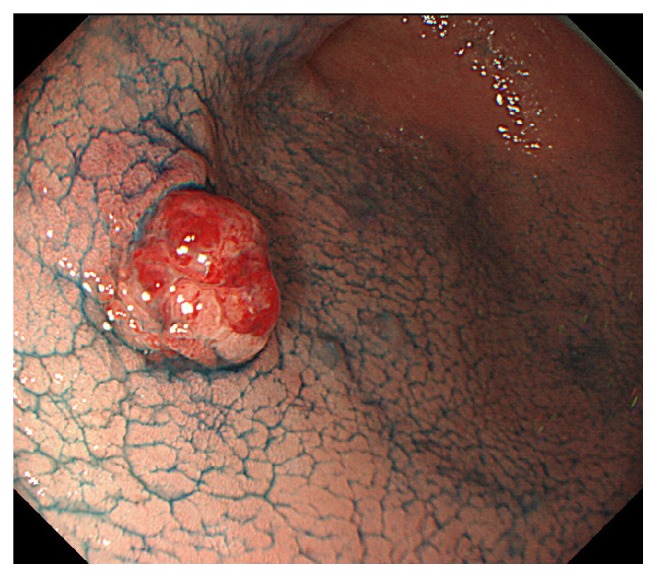
Gastroscopy revealed a 10-mm superficial elevated lesion with a slight central depression (type 0-IIa+IIc).

A 73-year-old woman was admitted to our hospital after a gastric tumor was identified by gastroscopy following medical examination. Gastroscopy revealed a 10-mm diameter, superficial elevated lesion with a slight central depression (type 0-IIa+IIc; Figure 1) at anterior wall of lower gastric body. Biopsy findings indicated a diagnosis of a poorly differentiated carcinoma. No lymph node or distant metastases was identified via computed tomography; however, submucosal invasion was suspected and, hence, an endoscopic submucosal dissection was performed for a therapeutic diagnosis.

**Figure 2:**
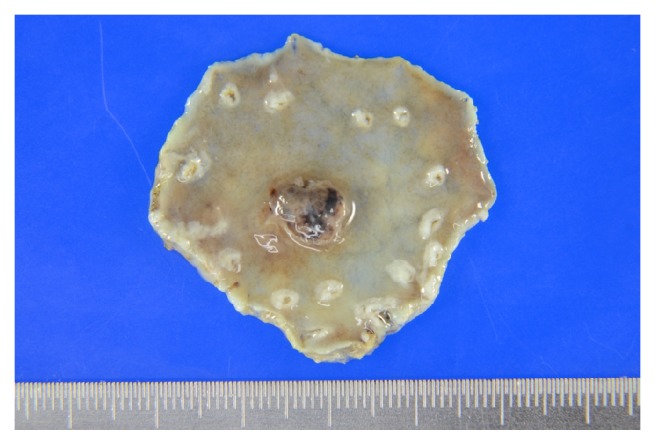
Gross findings of the formalin-fixed endoscopically resected specimen. The tumor was 10 × 8 mm in size, well-circumscribed, and accompanied by hemorrhage.

**Figure 3:**
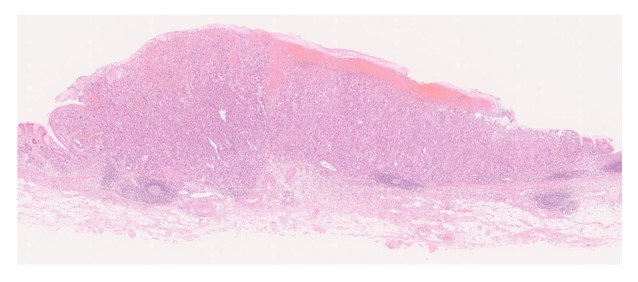
Low-magnification microscopic findings. Note the invasion of the submucosal layer.

**Figure 4:**
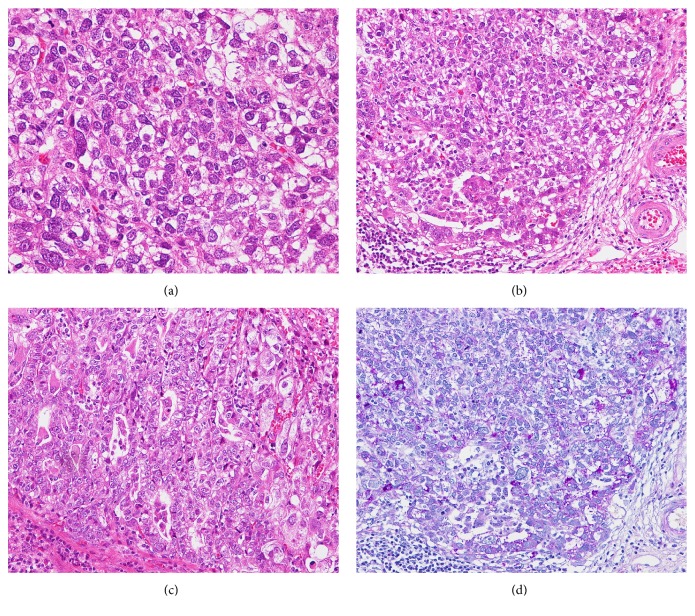
High-magnification microscopic findings. (a) Cuboidal tumor cells with round to irregular-shaped nuclei, prominent nucleoli, and the characteristically clear cytoplasm. Hematoxylin–Eosin (HE) stain. (b) The tumor growth pattern was predominantly solid and trabecular. HE stain. (c) Some tubular growth formation was identified. HE stain. (d) Alcian blue and periodic acid-Schiff stain of the specimen, demonstrating abundant glycogen in the cytoplasm.

Macroscopically, the tumor was 10 × 8 mm in size, well-circumscribed, and accompanied by hemorrhage (Figure 2). Low-magnification microscopy confirmed the invasion of the submucosal layer (Figure 3), whereas at high-magnification microscopy revealed cuboidal tumor cells with round to irregular-shaped nuclei, a prominent nucleolus, and clear cytoplasm characteristic of GCED (Figure 4(a)). A predominantly solid and trabecular growth pattern with a small proportion of tubular formation was also identified (Figures 4(b) and 4(c)). The degree of nuclear atypia was severe, and cells with deformed nuclei or multinucleation were scattered. Mitosis was common, and atypical mitosis was also identified. Further findings included conspicuous stromal hemorrhage, abundant cytoplasmic glycogen (according to Alcian blue and periodic acid-Schiff staining (Figure 4(d))), absence of mucin, and lymphovascular invasion. No CA component, hepatoid carcinoma, yolk sac tumor, or other histological cell types were found in any section. The tumor was surrounded by atrophic pyloric mucosa without intestinal metaplasia, and* Helicobacter pylori* was absent.

**Figure 5:**
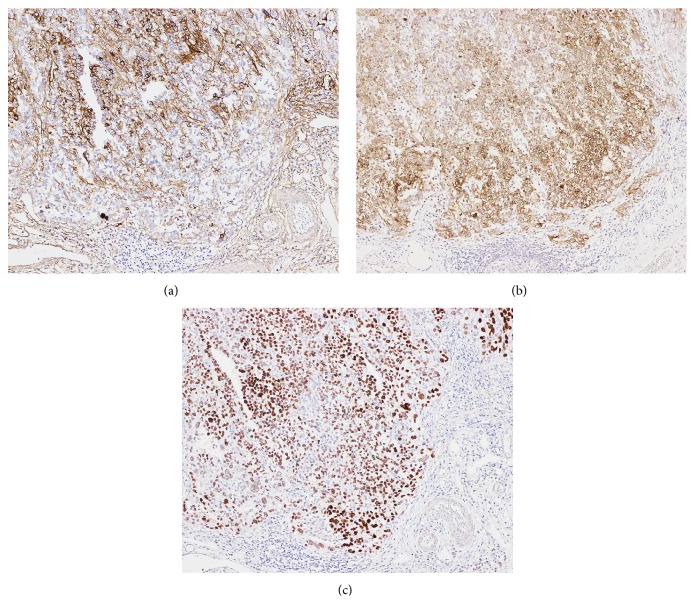
Immunohistochemical findings. The tumor cells were positive for the enteroblastic lineage biomarkers (a) AFP, (b) GPC3, and (c) SALL4.

Immunohistochemically, the tumor cells were positive for the enteroblastic lineage biomarkers AFP (rabbit polyclonal, 1:250; Dako, Glostrup, Denmark), GPC3 (clone 1G12, prediluted; Nichirei, Tokyo, Japan), and SALL4 (clone 6E3, 1:800; Abnova, Taipei, Taiwan) (Figures 5(a)–5(c)); they were negative for synaptophysin (clone 27G12, 1:100; Novocastra, Newcastle, UK) and HER2 (clone TAB250, 1:1; Zymed, San Francisco, CA, USA).

The chosen treatment was distal gastrectomy with lymph node dissection. The resected specimen indicated no lymph node metastasis and complete resection. Serum AFP level was normal after resection, although it was not examined before endoscopic submucosal dissection was performed.

## 3. Discussion

AFP-producing gastric carcinoma is a functionally defined variant of gastric carcinoma based on the function of tumor, and it falls into some histological types based on morphology of tumor. Reports state hepatoid carcinoma as the representative type along with GCED, tubular adenocarcinoma, papillary adenocarcinoma, poorly differentiated adenocarcinoma, and yolk sac tumor [1–9]. The term “GCED” was first used in 1994 by Matsunou et al. [4], although there were prior reports of AFP-producing gastric carcinoma with clear cytoplasm and glandular formation [10, 11]. Moreover, there are several published reports identifying GCED in particular, and, currently, AFP-negative cases are included in GCED in case of corresponding morphology and immunohistochemical positivity of other enteroblastic lineage markers (GPC3 or SALL4) [1–4, 12, 13]. The histogenesis of GCED has not been clarified, but it has been reported that AFP-producing gastric carcinoma often have mixed histologic types [5, 9–12]. Kinjo et al. hypothesized that CA in the mucosa differentiated into GCED and hepatoid carcinoma and acquired AFP production ability during the process of tumor invasion and proliferation; this was based on the histological findings, which indicated that the residual mucosal lesion was CA, which were intestinal type in many cases and/or GCED in almost all cases; however, hepatoid carcinoma was observed only in invasive lesions [3]. Two subsequent studies on GCED also demonstrated that the superficial mucosal layer was overlaid with CA, and GCED components existed only in the deeper part of the mucosa and/or submucosa [1, 2]. A genetic study on AFP-producing gastric carcinoma that included several cases of GCED supported this hypothesis, identifying the heterogeneous patterns of the loss of heterozygosity by comparing the mucosal lesion with the invasive lesion [7]. To the best of our knowledge, only three studies of GCED have measured the presence or absence of a residual mucosal lesion and a CA component on a case-by-case basis; only two of the 58 total patients had no CA component [1–3]. One case was that of a patient with a mucosal lesion showing purely enteroblastic differentiation, as seen here, whereas the other demonstrated serosal exposure with a hepatoid carcinoma component [3]. Therefore, early gastric cancer showing purely enteroblastic differentiation without a CA component appears to be very rare. This suggests that, mechanistically, GCED develops de novo in the mucosa, although the possibility that the CA components disappeared during the process of tumor invasion and proliferation cannot be ruled out.

In a previous study, lymphovascular invasion, lymph node metastasis, and liver metastasis were more frequently observed in GCED and AFP-producing gastric carcinoma than in CA [1, 2, 14, 15]. A study on endoscopically resected early-stage GCED found that GCED was more frequently associated with submucosal invasion and noncurative resection than with CA and that their depth tended to be underestimated [1]. Furthermore, a recent study suggests that a solid growth pattern is associated with a poor overall survival in GCED [14]. Notably, in the present patient, the typical primitive gut-like tubulopapillary growth pattern was limited, and the biopsy specimen was composed of almost all solid and trabecular structures; this made it difficult to make a diagnosis. Enteroblastic lineage markers (AFP, GPC3, and SALL4) are useful to make a diagnosis of GCED [1, 2]. To conclude, we reported on a very rare case of a patient with GCED, in whom differentiation was purely enteroblastic and the typical CA component was absent despite the presence of early cancer. This case suggests that GCED arises de novo in the mucosa. GCED is more aggressive compared with CA, and predicting the likelihood of submucosal invasion is more difficult. Thus, an accurate diagnosis is essential; pathologists should be aware of the possibility that GCED appears without CA in the biopsy specimens of early gastric cancer.
